# The In Vitro Inhibitory Effect of Sivelestat on Elastase Induced Collagen and Metallopeptidase Expression in Equine Endometrium

**DOI:** 10.3390/ani10050863

**Published:** 2020-05-16

**Authors:** Ana Amaral, Carina Fernandes, Maria Rosa Rebordão, Anna Szóstek-Mioduchowska, Karolina Lukasik, Barbara Gawronska-Kozak, Luís Telo da Gama, Dariusz J. Skarzynski, Graça Ferreira-Dias

**Affiliations:** 1 CIISA—Centre for Interdisciplinary Research in Animal Health, Faculty of Veterinary Medicine, University of Lisbon, 1300-477 Lisbon, Portugal; nita.amaral@gmail.com (A.A.); fachica@hotmail.com (C.F.); milorebordao@gmail.com (M.R.R.); ltgama@fmv.ulisboa.pt (L.T.d.G.); 2 Coimbra College of Agriculture, Polytechnic Institute of Coimbra, 3045-601 Coimbra, Portugal; 3 Institute of Animal Reproduction and Food Research, Polish Academy of Science,10-748 Olsztyn, Poland; a.szostek-mioduchowska@pan.olsztyn.pl (A.S.-M.); k.lukasik@pan.olsztyn.pl (K.L.); b.kozak@pan.olsztyn.pl (B.G.-K.); d.skarzynski@pan.olsztyn.pl (D.J.S.)

**Keywords:** endometrosis, mare, elastase, sivelestat, collagen, metallopeptidases, endometrium, neutrophil extracellular traps (NETs)

## Abstract

**Simple Summary:**

The protease elastase found in neutrophil extracellular traps appears to be associated with equine endometrial fibrosis by its stimulatory effect on extracellular matrix (ECM) components, leading to an increase in collagen relative abundance. Metallopeptidases (MMP-2 and -9) are enzymes involved in ECM remodeling. The modulation of elastase-induced deleterious effect on ECM and MMPs could be important for the prevention of fibrosis development. The selective inhibitor sivelestat is known to inhibit elastase activity. Our results showed that sivelestat inhibits the production of elastase-induced collagen in vitro by equine endometrial explants, and that MMP-2 and MMP-9 might be implicated in endometrium fibrotic response to elastase. By inhibiting elastase, it would be possible to prevent fibrosis development in mare’s endometrium.

**Abstract:**

Neutrophil extracellular traps (NETs) fight endometritis, and elastase (ELA), a protease found in NETs, might induce collagen type I (COL1) accumulation in equine endometrium. Metallopeptidases (MMPs) are involved in extracellular matrix balance. The aim was to evaluate the effects of ELA and sivelestat (selective elastase inhibitor) on MMP-2 and MMP-9 expression and gelatinolytic activity, as well as the potential inhibitory effect of sivelestat on ELA-induced COL1 in equine endometrium. Endometrial explants from follicular (FP) and mid-luteal (MLP) phases were treated for 24 or 48 h with ELA, sivelestat, and their combination. Transcripts of *COL1A2*, *MMP2*, and *MMP9* were evaluated by qPCR; COL1 protein relative abundance by Western blot, and MMP-2 and MMP-9 gelatinolytic activity by zymography. In response to ELA treatment, there was an increase in *MMP2* mRNA transcription (24 h) in active MMP-2 (48 h), both in FP, and in *MMP9* transcripts in FP (48 h) and MLP (24 h) (*p* < 0.05). Sivelestat inhibited ELA-induced *COL1A2* transcripts in FP (24 h) and MLP (24 h, 48 h) (*p* < 0.05). The sivelestat inhibitory effect was detected in *MMP9* transcripts in FP at 48 h (*p* < 0.05), but proteases activity was unchanged. Thus, MMP-2 and MMP-9 might be implicated in endometrium fibrotic response to ELA. In mare endometrium, sivelestat may decrease ELA-induced COL1 deposition and hinder endometrosis development.

## 1. Background

After breeding, mares develop a transient physiological endometritis, which resolves shortly in healthy uteri. The semen-induced uterine inflammation is characterized by a fast arrival of neutrophils into the uterine lumen [1,2]. The influx of inflammatory cells in the mare’s uterus empowers the inflammatory reaction, resulting in the removal of unnecessary spermatozoa, contaminating bacteria, and debris [3,4]. In addition, active neutrophils at the inflammation site also release their DNA and cytoplasm proteins to the extracellular environment, such as histones, and proteases as elastase (ELA), cathepsin G (CAT), and myeloperoxidase (MPO), forming neutrophil extracellular traps (NETs) [5,6]. Equine neutrophils produce NETs in the mare endometrium in the presence of *Escherichia coli* and *Streptococcus equi* subspecies *zooepidemicus* [7], or in contact with equine semen [8,9]. However, the proteases found in NETs might also induce a pro-fibrotic response in the endometrium of mares susceptible to chronic endometritis (endometrosis), characterized by the accumulation of collagen type I (COL1), which may link these proteases to endometrosis pathogenesis [10,11].

After tissue injury, for extracellular matrix (ECM) reorganization, and especially in the presence of continuous stimuli, the parenchymal tissue is replaced by connective tissue components, such as interstitial COL1 [12]. If the balance between ECM synthesis and degradation fails, it leads to fibrosis and to an increase in ECM components’ deposition and/or a reduction of its degradation. Metallopeptidases (MMPs) are proteases involved in ECM balance maintenance. Among them, MMP-2 and MMP-9 are enzymes that denature collagens (gelatins) and other ECM substrates [13]. However, it has been documented that MMPs can have both stimulatory or inhibitory effects in fibrosis and can act differently among organs [14]. MMP-2 and MMP-9 are also related to the migration of fibrocytes in idiopathic pulmonary fibrosis [15], as well as to myofibroblast activation in vascular fibrosis [16]. In the liver and kidney, MMP-2 appears to have an anti-fibrotic effect and MMP-9 has a pro-fibrotic role [14]. In fact, in the early stages of fibrosis in hepatic tissue, MMP-9 is capable of activating the TGFβ1 pathway, while in the later stages of established fibrosis MMP-2 reduced COL1 relative abundance [17]. It has also been suggested that, in pulmonary fibrosis, MMP-9 is linked to inflammatory-induced tissue remodeling, while MMP-2 may be associated with impaired tissue remodeling, leading to abnormal collagen deposition and interstitial fibrosis [18]. Our studies showed that the endometrial expression of MMPs and their tissue inhibitors (TIMPs) is altered at the different stages of endometrosis, and in response to interleukins [19,20].

Elastase is a serine protease that has been reported to be increased in neutrophils retrieved from the sputum of cystic fibrosis patients [21], and to induce in vitro lung fibroblast proliferation and myofibroblast differentiation [22]. Recently, we have found that ELA induced *COL1A2* mRNA transcripts [10,11] and COL1 relative abundance [10] in equine endometrium explants, suggesting ELA´s involvement in the development of equine endometrosis.

The use of sivelestat sodium salt (SIV), which is a selective inhibitor of ELA retrieved from neutrophils, has shown beneficial effects on fibrosis impairment, either during in vitro studies or in clinical trials. Sivelestat has been reported to reduce pulmonary deposition of COL and fibrosis in mice [23], and to diminish the in vitro *COL1A2* transcription in equine endometrium [11]. In addition, SIV administration in human patients with acute lung injury has improved their clinical condition and prognosis [24,25]. Altogether, the inhibition of the pro-fibrotic effects of ELA by SIV in several fibrotic diseases in a number species substantiate the use of SIV as a potential therapeutic approach for equine endometrosis. Therefore, the rationale for this study was to evaluate whether COL1 production could be restrained when mare endometrium was challenged by the protease ELA found in NETs. Thus, the aim of this in vitro study was to evaluate the inhibitory effect of SIV on ELA induced COL1 protein relative abundance in equine endometrial explants, and the effect of ELA and SIV on the expression and activity of MMP-2 and MMP-9.

## 2. Materials and Methods

### 2.1. Animals and Tissue Collection

The mares used in the present study were healthy, as determined by official veterinary inspection, and presented ovarian cyclicity. These mares were used for meat production for human consumption. They were handled and euthanized at horse abattoirs in Poland, according to the European (EFSA, AHAW/04–027) mandates. From April 2017 to September 2018, uteri were retrieved post-mortem from follicular phase (FP; *n* = 8) and mid-luteal phase (MLP; *n* = 7) mares. Prior to euthanasia, peripheral blood samples from the jugular vein were collected into heparinized tubes (Monovettes, Sardtedt, Numbrecht, Germany). Progesterone (P4) plasma concentrations were further determined to confirm the phase of the estrous cycle, firstly based on ovarian structures evaluation immediately after slaughter, as previously described [26]. Briefly, presence of a follicle >35 mm diameter, absence of an active corpus luteum (CL), and plasma P4 concentration <1 ng/mL were characteristic of mares in the FP. In contrast, in the MLP, a well-developed CL was associated with follicles between 15 and 20 mm diameter and a plasma P4 concentration >6 ng/mL [26]. The uteri were immersed in ice-cold Dulbecco’s modified Eagle’s medium (DMEM) F-12 Ham medium (D/F medium; 1:1 (*v*/*v*); D-2960; Sigma, St Louis, MO, USA), supplemented with 100 µg/mL streptomycin (S9137; Sigma), 100 IU/mL penicillin (P3032; Sigma), and 2 µg /mL amphotericin (A2942; Sigma). After collection, uteri and blood were transported on ice to the laboratory, within 1 h. All the collected uteri were confirmed for the absence of endometritis, as previously described [10,27].

### 2.2. In Vitro Endometrial Explant Culture

The uteri were washed in phosphate-buffered saline (PBS) with 100 µg/mL streptomycin (S9137; Sigma) and 100 IU/mL penicillin (P3032; Sigma), and the ipsilateral horn to the active ovary was open and strips of endometrium were detached from myometrium using scissors. Two endometrial samples were immersed in 4% buffered formaldehyde for histological evaluation and endometrial classification. Endometria were histologically graded according to Kenney and Doig´s classification [28], based on the extent of inflammation and/or fibrosis, as category I, IIA, IIB, or III, corresponding to minimum, mild, moderate, or severe lesions of endometrial fibrosis, respectively. In order to group and normalize the samples, only mare endometria classified as grade IIA or IIB were considered in this study. Thereby, the variation due to endometrium category was excluded from this experiment.

The endometrial strips were placed in phosphate-buffered saline (PBS) with 100 µg/mL streptomycin (S9137; Sigma) and 100 IU/mL penicillin (P3032; Sigma) in a petri dish on ice. Endometrial explants (20–30 mg/well) from FP or MLP were placed in 1 mL of DMEM culture medium supplemented with 0.1% (*w*/*v*) bovine serum albumin (BSA; 735078; Roche Diagnostics, Mannheim, Germany), 100 µg/mL streptomycin (S9137; Sigma), 100 IU/mL penicillin (P3032; Sigma), and 2 µg /mL amphotericin (A2942; Sigma), in a single well in a 24-well sterile cell culture plate (Eppendorf, #0030 722.116) for 1 h, at 37 °C, 5% CO_2_ in a humidified atmosphere (Biosafe Eco-Integra Biosciences, Chur, Switzerland) with gentle shaking (150 rpm), as described [10]. After 1 h treatment, the culture medium was replaced, and explants were further treated for 24 h or 48 h, as follows: (i) vehicle (control)—culture medium alone; (ii) elastase (ELA; 0.5 µg/mL; A6959, Applichem GmbH, Germany); (iii) ELA inhibitor: sivelestat sodium salt (SIV; 10 µg/mL; sc-361359; Santa Cruz Biotechnology, USA); (iv) ELA (0.5 µg/mL) + SIV (10 µg/mL); (v) transforming growth factor beta β1 (TGFβ1; 10 ng/mL; GF111; Merck, Darmstadt, Germany), used as a positive control for the assessment of fibrogenic capacity on endometrial explants, as established before [10,29]; or (vi) oxytocin (OXT; 10^−7^ M), a positive control for prostaglandin (PG) secretion—validation of proper secretory function of endometrial explants in long-term culture [30,31]. The ELA inhibitor (SIV) was added at the time of culture medium replacement, while proteases present in NETs were only added 1 h later, to give the inhibitor time to bind. Each treatment was applied in quadruplicate. After incubation, explants were placed in RNAlater^®^ (R901, Sigma) at 4 °C, overnight. Explants and conditioned culture media were stored at −80 °C. The culture media for PG determination was collected into a 1% stabilizer solution of 0.3 M ethylenediaminetetraacetic acid (EDTA; E5134, Sigma) and 1% aspirin (A2093; Sigma) to prevent PG degradation.

The ELA dose-response assessment was based on a previous study where 0.5 µg/mL proved to induce the release of TGFβ1, a fibrotic marker, and production of COL1 in equine endometrial explants [10]. In addition, the concentration of ELA used is within the range of the concentrations found in physiological and inflammatory processes and has been used in other in vitro assays [32]. In order to determine the most adequate concentration of SIV, a dose-response trial was carried out based on previous in vitro studies that used SIV (0.01, 0.1, 1, 10, and 100 µg/mL) [11,33]. In the preliminary work, 10 µg/mL was the optimal concentration of SIV, which was able to inhibit ELA by reducing *COL1A2* transcripts in mare endometrium [11]. This SIV concentration provoked an inhibitory effect on *COL1A2* transcription that remained for 24 h, but after the 48 h treatment, this effect was reduced. Therefore, 10 µg/mL of SIV was added again to the culture medium at the end of the 24 h treatment, with explants undergoing a total of 48 h of treatment.

### 2.3. Viability of Endometrial Explants

The viability of endometrial samples was determined based on PG secretion in conditioned culture medium and on lactate dehydrogenase (LDH) activity. Prostaglandin F_2α_ in culture medium was determined by an enzyme immunoassay kit (PGF2α ELISA kit—ADI-901-001, Enzo, USA and ELISA kit—ADI-901-069, Enzo), according to the manufacturer’s instructions. The standard curve ranged from 3 to 50,000 pg/mL and the intra-and inter-assay coefficients of variance (CVs) were 5.9% and 4.3%, respectively. The outputs of PGF_2α_ were used to check the secretory capacity of the non-treated and OXT-treated tissues, suggesting that the endometrial explants contain functional cells [30,31]. The LDH activity was assessed by a colorimetric assay kit (ab102526, Abcam, UK) according to the manufacturer’s procedures. The enzyme LDH converts pyruvate into lactate with concomitant inter-conversion of NADH, whose concentration was measured. Extracellular LDH activity was measured in explant conditioned culture media (1 h, 24 h, and 48 h incubation) after a 1:100 dilution in the kit assay buffer. For the measurement of intracellular LDH, 10 mg of the incubated explants (1 h, 24 h, and 48 h) was homogenized using a disruptor (TissueLyser II; Qiagen, Madrid, Spain) in 250 µL kit assay buffer and diluted 1:200 times in the same buffer. The LDH activity was read spectrophotometrically (FLUOstar OPTIMA Microplate Reader; BMG Labtech; Ortenberg; Germany) in a kinetic mode at 450 nm wavelength, at 37 °C, for 1 h. Since the point at which the cell membrane is damaged, and LDH is released to the extracellular environment, explant viability was calculated from the quotient of the intracellular LDH activity and the total activity (extracellular plus intracellular LDH) [34].

### 2.4. Quantitative Real-Time Polymerase Chain Reaction (qPCR)

Total RNA was extracted using TRI Reagent^®^ (T9424; Sigma) according to the manufacturer’s instructions. The quantification of RNA was performed using the Nanodrop system (ND 200C; Fisher Scientific, Hamton, PA, USA) and its quality was assessed by visualization of 28S and 18S rRNA bands after electrophoresis through a 1.5% agarose gel and red staining (41,003; Biotium, Hayward, CA, USA). Reverse transcription was carried out with M-MLV reverse transcriptase enzyme (M5313; Promega; Madison, USA) from 1000 ng total RNA in a 20 µL reaction volume using oligo(dT) primer (C1101; Promega).

Specific primers for *COL1A2, MMP2, MMP9*, and the reference gene ribosomal protein L32 (*RPL32*) were previously designed by us using Primer3 Software and Primer Express (Applied Biosystems, Foster City, CA, USA) [10]. The primers used are listed in Table 1. The genes glyceraldehyde 3-phosphate dehydrogenase (*GAPDH*), succinate dehydrogenase A complex, subunit A, flavoprotein (*SDHA*), beta-2-microglobulin (*B2M*), and *RPL32* were tested to determine which should be used as reference gene. In PCRs with efficiencies approaching 100%, the amount of internal reference gene relative to a calibrator (fold change between two Ct values) is given by the following equation: Fold difference = 2^−ΔCt^. At a reaction efficiency of 100%, one cycle (expressed as Ct in qPCR) corresponds to a twofold change [35]. As *RPL32* was the most stable internal control gene in our experimental conditions (less than twofold changes between different biological conditions) [35], it was used as the reference gene throughout the study.

After primer concentrations optimization in a StepOnePlus™ Real-Time PCR System (Applied Biosystems, Warrington, UK), target and reference genes were run simultaneously, and all the reactions were performed in duplicate on a 96-well plate (4306737; Applied Biosystems). Products of PCR were run on a 2.5% agarose gel to confirm specificity, and relative mRNA data were quantified as described [10,36].

### 2.5. Western Blot Analysis

Relative protein abundance of COL1 was assessed by Western blot using a stain-free total protein loading control. The tryptophan present in proteins produces an ultraviolet (UV) reaction with trihalo compounds present in 2,2,2-trichloroethanol (TCE; 808610; Merck) used to stain acrylamide gels, which can be visualized as a fluorescent signal in a transilluminator [37,38]. Endometrial explants were minced and placed on ice-cold RIPA buffer (50 mM Tris-HCl, pH 7.4, 50 mM EDTA, 150 mM NaCl, and 1% Triton X-100) supplemented with a protease inhibitor (cOmplete Mini Protease Inhibitor Cocktail Tablets, 1 tablet per 10 mL of buffer; Roche, Basel, Switzerland) and briefly disrupted (TissueLyser II, Qiagen). After protein extraction, Bradford reagent (500-0006; Bio-Rad, Hercules, CA, USA) was used for determination of protein concentration. Afterwards, 30 µg of protein in 2× Laemmli Loading Buffer (62.5 mM Tris-HCl, pH 6.8 containing 2% SDS, 25% glycerol, 0.01% bromophenol blue) was prepared. Then, the reducing agent DTT was added fresh to obtain a final concentration of 50 mM. Denaturation of proteins was accomplished by heating at 95 °C for 5 min and then cooling on ice for 10 min. The samples were loaded on an 8% acrylamide gel (MB04501; Nzytech, Lisbon, Portugal) with 0.5% (*v*/*v*) TCE incorporated in gel [37] using a Mini-PROTEAN^®^ Vertical Tetra Cell system (Bio-Rad). Just before transfer to a nitrocellulose membrane (GE10600001; Amersham™ Protran^®^ Western blotting membranes, nitrocellulose pore size 0.2 μm, roll W × L 300 mm × 4 m; GE Healthcare; Chicago, IL, USA), the gels were exposed for 1 min to UV light at ChemiDoc XRS + System (Bio-Rad). After transfer (Mini-Trans^®^ Blot, Bio-Rad), the membranes were also exposed for 1 min to UV light to obtain the final image to use in the normalization channel. An image of gels after transfer was also kept, ensuring that the transfer occurred effectively. The membranes were incubated overnight, at 4 °C with the primary antibody against COL1 (1:1,000 diluted; 20121; Novotec, Lyon, France), as previously described and validated [10]. Afterwards, the membranes were incubated with the secondary antibody horseradish peroxidase (HRP)-conjugated anti-rabbit (1:20,000 diluted; P0448; DakoCytomation, Carpinteria, CA, USA) for 1.5 h at room temperature. The COL1 protein relative abundance was visualized using luminol enhanced chemiluminescence (Super Signal West Pico, 34077; Thermo Scientific, Waltham, MA, USA) and image acquisition was performed by ChemiDoc XRS + System (Bio-Rad). A standard sample (30 µg) of mixed endometrial explants was loaded in all gels in a single lane, in order to normalize all bands in the same membrane and to compare bands between membranes. Relative abundance of COL1 protein was analyzed using Image Lab 6.0 (Bio-Rad) software and by creating a multichannel protocol, which allowed the lanes’ detection in stain-free total protein membrane image and bands’ detection on chemiluminescence image after incubation with the antibodies. The software calculated the normalization factor and volume of target protein, and the values were adjusted for variation in the protein load [39]. The use of a protein loading control has been questioned owing to its possible instability in certain samples [38], variations ascribed to experimental conditions, and the saturation of the chemiluminescent signal from the loading control proteins [40]. Some studies refer to these proteins as not being suitable as a loading control [38,40]. Therefore, a better solution is to use a stain-free total protein loading control as it measures the real amount of protein loaded and considers the real differences among samples [39]. In our preliminary studies, using equine endometrial tissue, this blot normalization technique was shown to produce cleaner images providing an improving normalization (data not shown).

### 2.6. Zymography

The most simple, sensitive, and effective method to analyze MMPs is zymography. It allows the proteins to separate by electrophoresis under denaturing and non-reducing conditions in a polyacrylamide gel containing gelatin to detect proteases, namely gelatinases MMP-2 and MMP-9, which degrade gelatin. As in Western blot analysis, zymography normalization was done using a stain-free total protein loading control. The protein content of culture medium supernatant from the explants cultured was measured using the Bradford method. The general protocol followed was previously described [41]. Thus, 40 µg of protein in 2× sample buffer (62.5 mM Tris-HCl pH 6.8, 25% glycerol, 4% SDS, and 0.01% bromophenol blue) was loaded, without heating or reduction, to an 8% polyacrylamide gel (MB04501; Nzytech) containing 0.1% gelatin and 0.1% SDS. To verify MMPs’ molecular weight, MMP-2 and MMP-9 standards were loaded (Recombinant Human MMP2 Protein, CF -902-MP-010 and Recombinant Human MMP-9 Western Blot Standard Protein—WBC018; R&D Systems, Minneapolis, MN, USA) in all gels. SDS-PAGE electrophoresis was conducted in Mini-PROTEAN^®^ Vertical Tetra Cell system (Bio-Rad). The gels were then washed with 2.5% Triton X-100 for 40 min and incubated in the development solution (50 mM Tris–HCl buffer pH 7.5, 200 mM NaCl, 0.02% Triton X-100, and 5 mM CaCl_2_) for 16 h at 37 °C. After that, gels were incubated in 10% (*v*/*v*) TCE in a 1:1 methanol/water mixture for 10 min. As TCE can inhibit gelatinases activity, it should not be incorporated in gels [42]. Thus, gels were exposed for 5 min to UV light at ChemiDoc XRS + System (Bio-Rad), and then washed in distilled water to remove the TCE solution before staining (50% methanol, 10% acetic acid, and 0.1% Coomassie brilliant blue) for 30 min, and destained in the same solution in the absence of dye, until clear bands were visible. In a way to normalize all lanes and bands in the same gel and compare each gel with all the gels obtained in the experiment, a standard sample (40 µg) of mixed culture medium was loaded in all gels in a single lane. Image Lab 6.0 (Bio-Rad) software was used to analyze MMP-2 and MMP-9 by creating a multichannel protocol, which enabled lane detection in stain-free total protein gel image, and band detection on Coomassie staining image. The software calculated the normalization factor and volume of target protein, and the values were adjusted for variation in the protein load. The use of a stain-free total protein normalization and Coomassie staining is a better way to normalize and overcome variations on the protein loaded in each sample. Besides, this normalization method avoids variations between different experimental conditions and between gels [42].

### 2.7. Statistical Analysis

Statistical analysis of the viability data and TGFβ1 fibrogenic assay was performed using GraphPAD PRISM (Version 6.00, 253 GraphPad Software, San Diego, CA, USA). One-way analysis of variance (ANOVA) followed by Tukey’s multiple comparisons test was used to compare endometrial explants viability (PGF_2α_ concentration and LDH activity assay), and the effect of TGFβ1 treatment. These data are shown as mean ± SEM and the results were considered significant at *p* < 0.05.

The response variables evaluated in the experimental work were *COL1A2*, *MMP2*, and *MMP9* transcription measured by qPCR; COL1 protein relative abundance by Western blot; as well as MMP-2 and MMP-9 activity evaluated by zymography in both pro- and active forms. Compliance with normality after various transformations was assessed visually and using the Kolmogorov–Smirnov test in Proc Univariate function of SAS v. 9.4 (SAS Institute Inc., Cary, NC, USA). As many of these variables did not have a normal distribution, the square root and logarithmic transformation were tested, and the best transformation for a given variable was chosen for further analysis. In a preliminary analysis, each transformed response variable was analyzed with the PROC GLM of SAS, as a function of the various treatments that resulted from the combination of the use of ELA, use of SIV, estrous cycle phase, and time of treatment, for a total of 16 treatment combinations. The least square means for the various treatment combinations were compared with the PDIFF option of PROC GLM, assuming *p* < 0.05 as the threshold of significance, and the means were back transformed to the original scale for graphical presentation. In a second analysis, the factorial nature of the treatment combinations was evaluated, by considering the main effects of the factors above plus their two-, three-, and four-way interactions, allowing the comparison of specific treatment combinations. The results of COL1 relative abundance protein, *COL1A2*, *MMP2*, and *MMP9* mRNA are shown as median with interquartile range. The MMP-2 and MMP-9 gelatinolytic activity results are shown as least square means ± SEM. The graphs were performed using GraphPAD PRISM. Back-transformed SEM are presented as 95% confidence interval.

## 3. Results

### 3.1. Validation of the Viability of Long-Term Endometrial Explant Culture

A preliminary experiment aimed to verify whether COL1 increases when the endometrial explants are exposed to TGFβ1, a fibrogenic agent. Treatment with TGFβ1 increased *COL1A2* transcription at both phases and times of treatment (FP: 24 h—*p* < 0.0001, 48 h—*p* < 0.001; MLP: 24 h—*p* < 0.05, 48 h—*p* < 0.01; Table 2), and augmented COL1 protein relative abundance in FP at 24 h (*p* < 0.001) and MLP at 24 and 48 h (*p* < 0.001; Table 2).

The viability of endometrial explants determined by LDH activity after 1 h, 24 h, or 48 h incubation is listed in Table 3. Differences were found between 1 h and 48 h, and between 24 h and 48 h incubation (*p* < 0.001; Table 3). The results were independent of estrous cycle phase.

In addition, PGF_2α_ secretion by endometrial explants after treatment with OXT increased compared with non-treated tissues at 24 h (*p* > 0.01) and 48 h (*p* > 0.05; Table 4). These results were independent of estrous cycle phase.

There were significant interactions between treatments, time of treatment, and estrous cycle phase. All data are shown in Supplementary Table S1.

### 3.2. Inhibitory Effect of Sivelestat on ELA-Induced COL1

**Figure 1:**
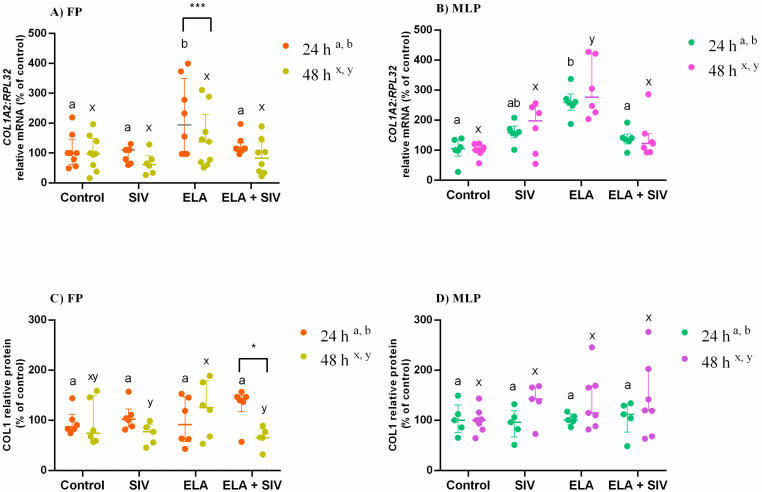
Relative collagen type I (*COL1A2*) mRNA transcription (**A**,**B**) and protein (COL1) relative abundance (**C**,**D**) in follicular phase (FP) and mid-luteal phase (MLP) mare endometrial explants treated for 24 or 48 h with medium alone (control), elastase (ELA: 0.5 μg/mL), sivelestat (SIV: 10 μg/mL), or ELA (0.5 μg/mL) + SIV (10 μg/mL). Data are shown as median with interquartile range. Results were considered significant at *p* < 0.05. Different superscript letters indicate significant differences between treatments within each treatment time (a,b—24 h; x,y—48 h). Asterisks indicate statistical differences between times of treatment for the same treatment (* *p* < 0.05; *** *p* < 0.001).

Endometrial explants treated with ELA increased *COL1A2* mRNA transcription in FP after 24 h (*p* < 0.0001; Figure 1A), and in MLP after 24 h (*p* < 0.01; Figure 1B) and 48 h (*p* < 0.0001; Figure 1B), compared with the respective control group. However, the combination of ELA and SIV reduced *COL1A2* mRNA, when related to the respective ELA-treated group (FP 24 h: *p* < 0.01; MLP 24 h: *p* < 0.05; MLP 48 h: *p* < 0.001; Figure 1A, B). In ELA-treated explants, *COL1A2* transcripts also increased when compared with the SIV-treated group in FP at 24 h (*p* < 0.0001, Figure 1A), and in MLP at 48 h (*p* < 0.01; Figure 1B). In addition, in FP endometrium treated with ELA for 48 h, COL1 protein relative abundance increased when compared with the SIV-treated group and ELA + SIV-treated group (*p* < 0.01; Figure 1C; Supplementary Material Figure S1).

In addition, ELA highly stimulated *COL1A2* transcripts at 24 h of treatment in FP mare endometria, when compared with the 48 h treatment (Figure 1A). In addition, at 48 h, the inhibitory effect of SIV on ELA induced-COL1 protein relative abundance in FP explants was higher compared with 24 h treatment (Figure 1C; Supplementary Material Figure S1).

The differences found between estrous cycle phases (FLP vs. MLP) within each treatment and treatment times are listed in Supplementary Table S2.

### 3.3. The Effect of ELA and SIV on MMP Expression

**Figure 2:**
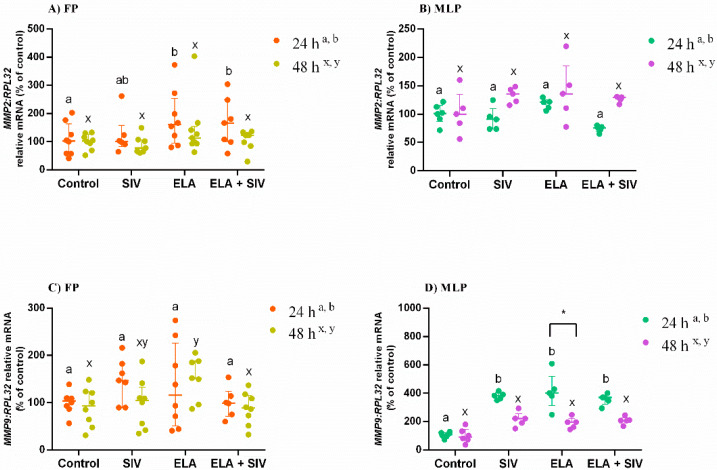
Relative mRNA transcription of matrix metallopeptidase 2 (*MMP2*) (**A**,**B**) and *MMP9* (**C**,**D**) in follicular phase (FP) and mid-luteal phase (MLP) mare endometrial explants treated for 24 or 48 h with medium alone (control), elastase (ELA: 0.5 μg/mL), sivelestat (SIV: 10 μg/mL), or ELA (0.5 μg/mL) + SIV (10 μg/mL). Data are shown as median with interquartile range. Results were considered significant at *p* < 0.05. Different superscript letters indicate significant differences between treatments within each treatment time (a,b—24 h; x,y—48 h). Asterisks indicate statistical differences between times of treatment for the same treatment (* *p* < 0.05).

Transcription levels of *MMP2* mRNA in endometrial explants were augmented in FP at 24 h with ELA and ELA + SIV- treated group compared with control (*p* < 0.01; *p* < 0.05 respectively; Figure 2A).

The transcripts of *MMP9* were upregulated in FP explants treated with ELA for 48 h, when compared with control (*p* < 0.05; Figure 2C). However, treatment with the combination of ELA + SIV reduced *MMP9,* when compared with the respective ELA-treated group (*p* < 0.05; Figure 2C). In MLP endometria, all treatments upregulated *MMP9* mRNA at 24 h (*p* < 0.01; Figure 2D). The transcripts of *MMP9* in ELA-treated explants were increased at 24 h in MLP with respect to 48 h in the same estrous cycle phase (Figure 2D).

**Figure 3:**
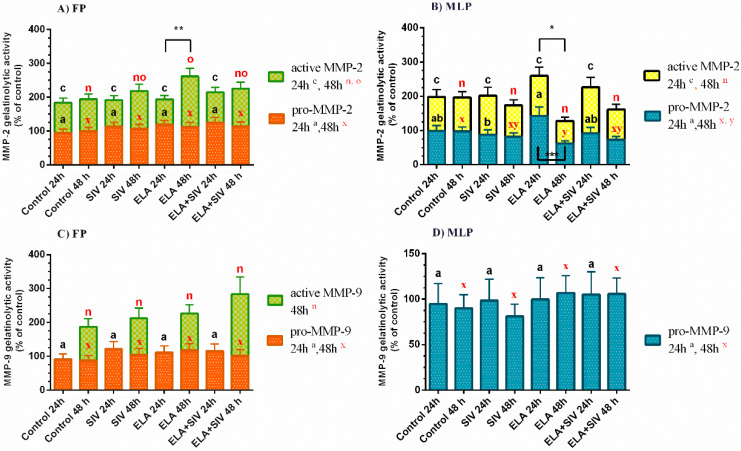
Relative gelatinolytic activities of MMP-2 (**A**,**B**) and MMP-9 (**C**,**D**) in follicular phase (FP) and mid-luteal phase (MLP) mare endometrial explants treated for 24 or 48 h with medium alone (control), elastase (ELA: 0.5 μg/mL), sivelestat (SIV: 10 μg/mL), or ELA (0.5 μg/mL) + SIV (10 μg/mL). All values are expressed as percentage of change from control (non-treated tissues). Bars represent least square means ± SEM and results were considered significant at *p* < 0.05. Different superscript letters indicate significant differences between treatments within each of treatment time. Asterisks indicate statistical differences between different treatment times for the same treatment, and for the same form of MMP (* *p* < 0.05; ** *p* < 0.01; *** *p* < 0.001).

The activity of pro-MMP-2 increased in MLP endometrial tissue treated for 24 h with ELA, when compared with SIV alone, while after 48 h, the activity subsided in explants treated with ELA compared with control (*p* < 0.05; Figure 3B). However, ELA increased the gelatinolytic activity of MMP-2 active form in FP endometrium after 48 h of treatment when compared with control (*p* < 0.05; Figure 3A; Supplementary Material Figure S1).

Differences between 24 and 48 h of treatment were found regarding the activity of pro- and active form of MMP-2 in MLP tissues. Thus, the 24 h treatment of endometrial explants with ELA induced the highest activity (Figure 3B). Nevertheless, it was after a 48 h treatment with ELA that the active form of MMP-2 in FP endometrial explants showed the highest activity (Figure 3C,D; Supplementary Material Figure S1).

In the active form of MMP-9, only FP explants treated for 48 h showed gelatinolytic activity (Figure 3A; Supplementary Material Figure S1).

The differences found between estrous cycle phases (FLP vs. MLP) within each treatment and treatment time are listed in Supplementary Table S2.

## 4. Discussion

The present study showed that ELA is capable of inducing *COL1A2* mRNA transcription by mare endometrial tissue in vitro, in both FP and MLP. This work, reinforced by our previous experiments, strengthens the hypothesis that ELA, as a pro-fibrotic protease, may play a role in the pathogenesis of endometrosis [10,11]. These data are in agreement with our previous study by Rebordão et al. [10] on endometria with moderate to severe lesions (Kenney and Doig IIB/III category) characteristics of endometrosis, where ELA was also capable of stimulating COL1 protein relative abundance. As a follow-up of those results, SIV was tested here as a specific ELA inhibitor.

In a porcine hepatectomy model of ischemia/reperfusion injury, SIV was reported to avoid organ failure by inhibiting vascular permeability and reducing cytokine production [43]. Studies on the use of SIV have been focused on the response to injury and inflammatory reactions, such as lipopolysaccharide-induced lung injury in rat lungs [44], reduced portal pressure associated with chronic liver diseases in mice [45], and bleomycin-induced pulmonary fibrosis in mice [23,46]. One should bear in mind that SIV has been largely reported as being administered to humans, mainly in acute lung diseases, to improve their clinical condition [47]. In fact, SIV acts by inhibiting the inflammatory cell recruitment and TGFβ1 activation in lungs, which is the putative mechanism for SIV modulatory action [23]. Therefore, we hypothesized that, inhibiting ELA, it would be possible to reduce COL1 deposition, and thus preventing fibrosis establishment at the course of endometrosis in mares. In fact, the inhibitory effect of SIV on ELA-induced *COL1A2* transcripts was observed in FP and MLP equine endometrium, reinforcing our preliminary results [11]. Thus, SIV might be a helpful inhibitor of ELA induced COL1 production in equine endometrium by reducing *COL1A2* gene transcription, and its use in fighting fibrosis may be considered as a putative therapeutic approach.

In the present work, the protein COL1 relative abundance did not follow the gene transcription pattern. The SIV inhibitory effect on ELA-induced COL1 protein relative abundance was only detected in FP explants treated for 48 h with ELA. Thus, it is likely that endometrium from FP, which is endogenously primed with estrogens, is more responsive to SIV treatment to impair COL production than the endometrium under the endogenous influence of progesterone in the MLP. It has been common to use mRNA transcription to predict the relative abundance of corresponding proteins, but the relative abundance of protein may not occur in proportion to their mRNA. Post-transcriptional, translational, and degradation regulation contributes to protein relative abundance at least as much as the transcription itself. The protein relative abundance should focus on the rates of protein production and turnover, and how this can change among different cellular conditions [48]. This model can fit in COL deposition in fibrosis, which is a chronic, progressive, and irreversible process. Possibly, the endometrium tries to prevent fibrosis establishment by increasing COL degradation as much as possible. Furthermore, as the high level of COL production needs 5000 times more mRNA than for the average protein, this process can take several days to induce an abundant level of COL protein, in contrast to the minutes needed to induce the synthesis of an average protein [49]. Despite high levels of transcription or translation, the most abundant proteins are often related to a slow translation, but very stable at a high final concentration [48]. Therefore, our experimental time window can be too short for the resultant COL protein production to be detected.

The turnover of COL and remodeling of ECM are regulated by MMPs, which are involved in protein degradation and in regulatory functions in inflammation and immunity [14]. The knowledge on MMP-2 and MMP-9 regulatory mechanisms facing a fibrotic stimulus is an important way to understand the pathogenesis of endometrosis. As a matter of fact, depending on the severity of endometrosis, the response to cytokine stimulation on MMP-2 and MMP-9 secretion by equine endometrial explants differed, which may associate them with endometrial microenvironment modifications that favor fibrosis establishment [19]. In the present study, endometria with mild/moderate endometrosis lesions (category IIA/IIB) showed different *MMP2* and *MMP9* mRNA levels and protein activity in response to ELA or SIV treatments, either alone or combined, depending on the treatment length. Those previous results [19] are consistent with ours, where MMPs’ expression seems to be different depending on estrous cycle phase and time of treatment. These findings suggest that hormonal changes and duration of the stimulus can affect the endometrial response. The protease ELA was capable of inducing *MMP9* mRNA transcription in FP endometrium at 48 h, and in MLP explants at 24 h. It has been reported that ELA activates pro-MMP-9 in cystic fibrosis in the lung [32]. In fact, the gelatinolytic activity of MMP-9 pro-enzyme was detected in equine endometrial explants, even though unchanged, while the active form was only observed in FP after a 48 h treatment, also unaltered. Regarding *MMP2* transcription, ELA treatment was also capable to induce a positive response in FP endometrium at 24 h, and in MMP-2 enzyme activity only at 48 h treatment time. It is worth mentioning that these enzymes are secreted to the extracellular environment or linked to cell membrane as inactive proenzymes [50], and their activity is regulated by transcription, protein production, and activation of latent enzymes [51]. This might explain the fact that the enzyme activity did not follow the gene transcription pattern. Nothnick [52] noted that *MMP9* transcripts may be present in high levels in the uterus of mice, but translation may be repressed, preventing protein and subsequent MMP-9 activity, with MMP-9 expression also being regulated by ovarian steroids. Taking our results into account, as the estrous cycle phase influenced the endometrial explant response to ELA and SIV treatment, it may be suggested that ovarian steroids in the mare can be implicated in MMPs’ secretion, as shown for mice [52]. Metallopeptidases, independent of their proteolytic function, seem to be associated with TGFβ1 activation [53,54,55,56], activation of other MMPs [57], myofibroblast differentiation [58], and cell proliferation [55,59], thus enhancing fibrosis. However, further studies are needed to confirm their action in the development of endometrial fibrosis in mare.

Despite decades of research on the treatment of endometrosis, no efficient therapy has been found. Even though claims have been made on the anecdotal use of intrauterine infusion of kerosene to treat endometrosis [60], no effect on the endometrium histopathology grade was noted [61]. In humans, for the treatment of pulmonary fibrotic conditions, SIV has been administered intravenously [24,47]. Likewise, knowledge transfer from the use of this ELA´s specific inhibitor for the treatment of fibrosis in humans could be applied to the horse. Thus, the novel findings from the present in vitro study might pave the way for testing the in vivo use of SIV in mares to prevent or hinder COL deposition in the endometrium. Specifically, in mares susceptible to post-breeding endometritis, associated with a prolonged inflammatory reaction and neutrophil influx into the uterus, SIV might be a potential therapeutic means to be tested in vivo against ELA induced fibrosis establishment. Therefore, this drug may be also beneficial to use in mares, either at the initial stages of fibrosis development, as well as in those showing full-fledged severe endometrosis. However, close caution should be taken, as SIV´s mechanisms of action, doses, as well pharmacokinetics (absorption, distribution, metabolism, excretion, and bioavailability) in horses are unknown. Moreover, different routes of administration, either intravenously or locally by uterine lavage, should be considered. NETs induced fibrosis development in mare endometrium is a complex process wherein many different proteases are involved [7,10]. Rather than ELA, we have shown that other proteases found in NETs, such as CAT and MPO, also induce COL1 protein relative abundance in equine endometrial explants [10]. As such, because COL deposition in mare endometrium exposed to NETs may result from the effect of many of their proteases, the use of a combination of different inhibitors of ELA, CAT, and MPO is a promising therapeutic approach to be considered.

## 5. Conclusions

The present data support the hypothesis that the protease ELA present in NETs is capable of inducing *COL1* mRNA transcription in equine endometrium and might be an important player in the regulatory cascade of the pathogenesis of endometrosis in mares. This fibrogenic action is inhibited by ELA selective inhibitor SIV, which may provoke a reduction in COL1 production by the mare endometrium. Moreover, further studies are needed to understand the cellular mechanisms and pathways leading to endometrosis, and the process in which MMP-2 and MMP-9 are involved. The complexity of equine endometrosis suggests that effective therapeutic interventions may require the administration of more than one agent, capable of inhibiting fibrosis in a nonspecific way. The promising results of the present work might be the basis for future development of putative therapeutic means to impair endometrosis. 

## Figures and Tables

**Table 1:** Primers used in quantitative real-time polymerase chain reaction (qPCR).

Gene (Accession Number)	Sequence 5′-3′	Amplicon
*COL1A2* (XM_001492939.3)	Forward: CAAGGGCATTAGGGGACACA	196
	Reverse: ACCCACACTTCCATCGCTTC	
*MMP2* (XM_001493281.2)	Forward: TCCCACTTTGATGACGACGA	115
	Reverse: TTGCCGTTGAAGAGGAAAGG	
*MMP9* (NM_001111302.1)	Forward: GCGGTAAGGTGCTGCTGTTC	177
	Reverse: GAAGCGGTCCTGGGAGAAGT	
*RPL32* (XM_001492042.6)	Forward: AGCCATCTACTCGGCGTCA	144
	Reverse: GTCAATGCCTCTGGGTTTCC	

*COL1A2*—collagen type 1 α2; *MMP2*—matrix metallopeptidase 2; *MMP9*—matrix metallopeptidase 9; *RPL32*—ribosomal protein L32.

**Table 2:** The effect of transforming growth factor beta β1 (TGFβ1) (10 ng/mL) on *COL1A2* mRNA transcription and COL1 protein relative abundance in follicular phase (FP) and mid-luteal phase (MLP) equine endometrial explants treated for 24 h or 48 h, relative to control (non-treated explants). Results are presented as fold-change means ± SEM. Different superscript letters indicate statistical differences between respective columns (within estrous cycle phases and times of treatment).

Estrous Cycle Phase	FP				MLP			
Time of Treatment	24 h		48 h		24 h		48 h	
Treatment	Control	TGFβ1 (10 ng/mL)	Control	TGFβ1 (10 ng/mL)	Control	TGFβ1 (10 ng/mL)	Control	TGFβ1 (10 ng/mL)
*COL1A2* transcription (fold increase)	0.66 ± 0.06 ^a^	0.97 ± 0.04 ^b^	1.02 ± 0.86 ^a^	1.82 ± 0.25 ^b^	1.00 ± 0.24 ^a^	2.75 ± 0.47 ^b^	1.00 ± 0.24 ^a^	3.86 ± 0.48 ^b^
COL1 protein (fold increase)	1.34 ± 0.05 ^a^	1.93 ± 0.12 ^b^	1.37 ± 0.05 ^a^	1.33 ± 0.05 ^a^	0.71 ± 0.54 ^a^	1.06 ± 0.01 ^b^	0.58 ± 0.02 ^a^	0.87 ± 0.004 ^b^

**Table 3:** Lactate dehydrogenase (LDH) activity measured in conditioned culture medium of equine endometrial explants after 1 h, 24 h, or 48 h incubation. Explants’ viability was calculated from the quotient of the intracellular LDH activity and the total activity (extracellular plus intracellular LDH). Results are presented as means ± SEM. Different superscript letters indicate statistical differences within time of incubation.

Time of Incubation	LDH Activity (%)
1 h	94.3 ± 0.9 ^a^
24 h	92.6 ± 0.5 ^a^
48 h	89.0 ± 0.6 ^b^

**Table 4:** The effect of oxytocin (OXT) on prostaglandin (PG) F_2α_ secretion in equine endometrial explants after 24 h or 48 h. Results are presented as means ± SEM. Different superscript letters indicate statistical differences within the different time of treatment.

Time of Treatment	24 h		48 h	
Treatment	Control	OXT (10^−7^ M)	Control	OXT (10^−7^ M)
PGF_2α_ secretion (ng/mg)	7.3 ± 0.8 ^a^	16.0 ± 1.3 ^b^	7.6 ± 0.9 ^a^	14.0 ± 3.2 ^b^

## Supplementary Materials

The following are available online at https://www.mdpi.com/2076-2615/10/5/863/s1, Figure S1: Representative panels of type I collagen western blotting and MMP-2 and MMP-9 zymograms, Table S1: Levels of significance for 2- and 3-way interactions between estrous cycle phases, treatment time, and elastase or sivelestat treatments in the evaluated variables, Table S2: Listed significant differences of the same treatments between the follicular phase and mid-luteal phase of the estrous cycle, within each treatment time.
