# Supplementary material for: The In Vitro Inhibitory Effect of Sivelestat on Elastase Induced Collagen and Metallopeptidase Expression in Equine Endometrium
Source: Animals (Basel). 2020 May 16;10(5):863. doi: 10.3390/ani10050863 (PMC7278485; doi:10.3390/ani10050863)
Supplement: Supplementary file 1 [file animals-10-00863-s001.pdf]

animals

MDPI

Supplementary Fig. S1

A –Follicular phase endometrium explants treated for 24h

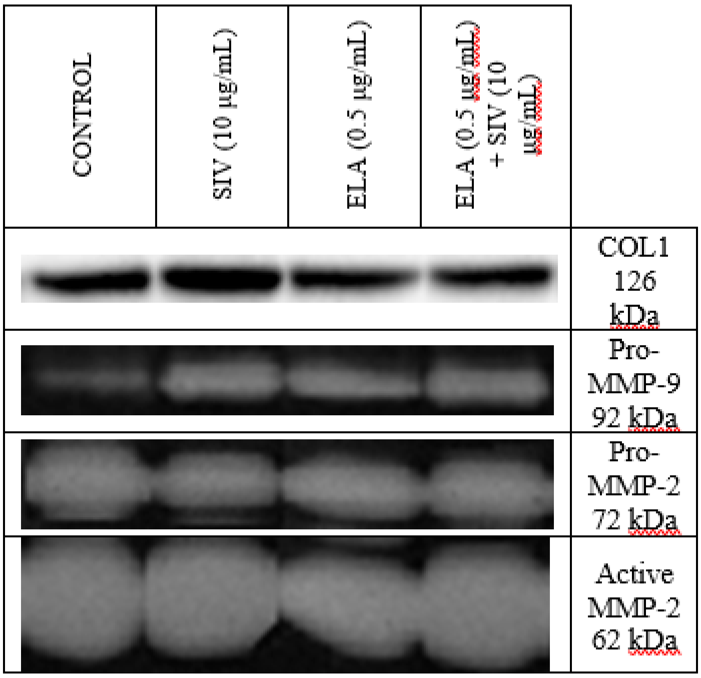

B – Follicular phase endometrium explants treated for 48h.

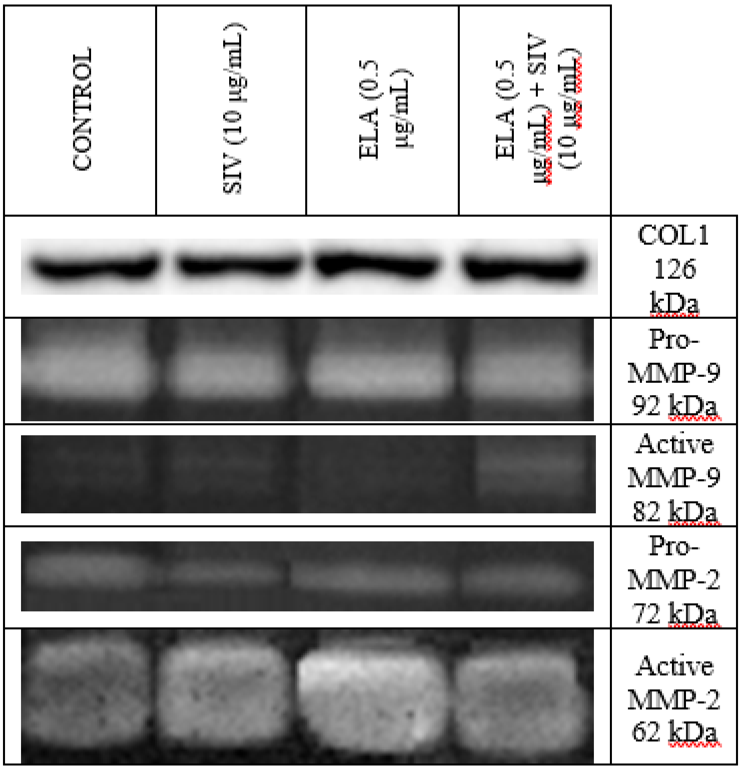

C – Mid-luteal phase endometrium explants treated for 24h

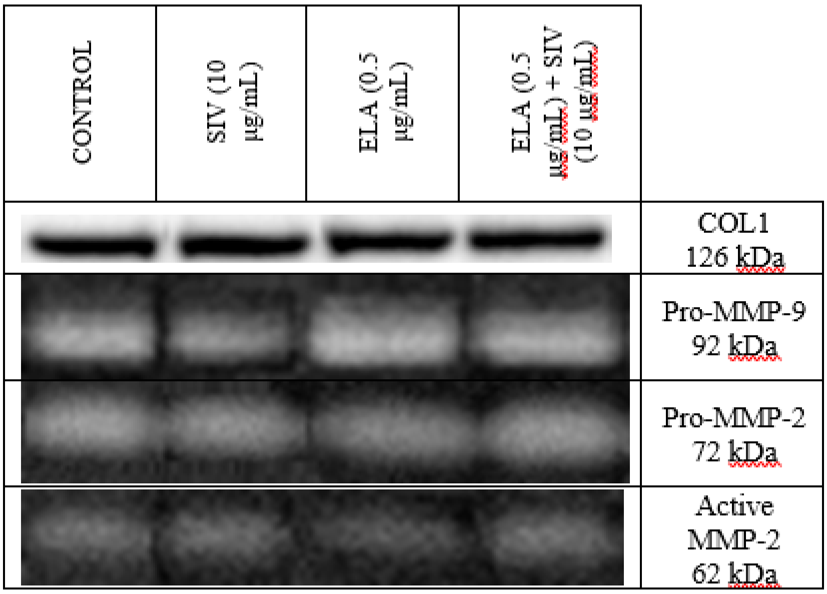

D – Mid-luteal phase endometrium explants treated for 48h

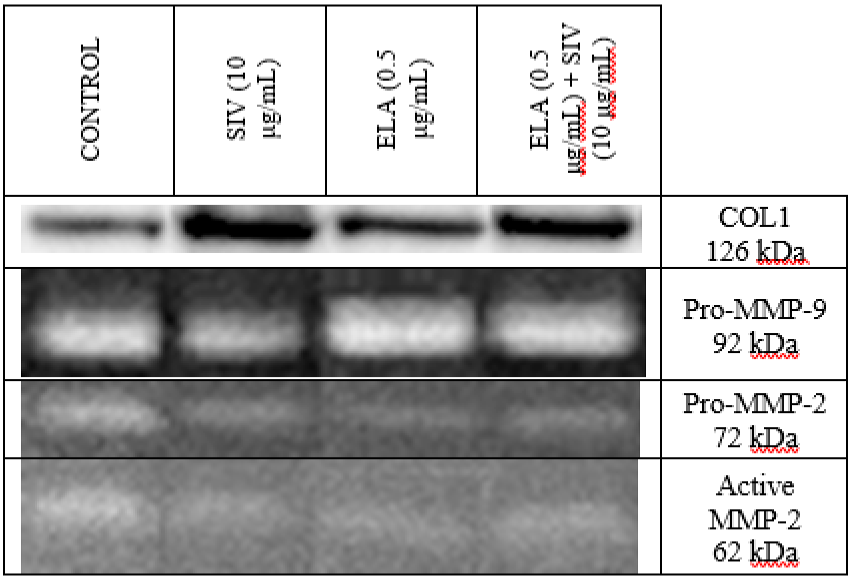

Figure S1. Representative panels of type I collagen (COL1) western blotting and pro- and active form of MMP-2 and MMP-9 zymograms in mare endometrium in follicular phase (FP) or mid-luteal phase (MLP) treated for 24h or 48h with elastase (ELA; 0.5 µg/mL), ELA inhibitor: sivelestat sodium salt (SIV; 10µg/mL) or ELA (0.5 µg/mL) + SIV (10µg/mL). **A** - 24h treatment of FP endometrium explants; **B** – 48h treatment of FP endometrium explants, **C** – 24h treatment of MLP endometrium explants; and **D** – 48h treatment of MLP endometrium explants.

Supplementary Tables

Table S1. Levels of significance (*P* values) for 2- and 3-way interactions between estrous cycle phases, treatment time, and elastase (ELA) or sivelestat (SIV) treatments in the analyses of relative transcript of target genes, COL1 protein relative abundance and gelatinolytic activity of MMP-2 and -9. The results were considered significant at *P* < 0.05 and are highlighted in yellow color.

Interaction	*COL1A2*	COL1	*MMP2*	Pro-MMP-2	Active MMP-2	*MMP9*	Pro-MMP-9	Active MMP-9
ELA x SIV	<0.0001	0.4035	0.1016	0.735	0.7368	0.0002	0.544	0.0728
ELA x treatment time	0.2078	0.6172	0.6266	0.0345	0.6855	0.8901	0.5405	
ELA x estrous cycle phase	0.9161	0.5926	0.1627	0.1991	0.4638	0.0342	0.8451	0.7948
SIV x treatment time	0.9928	0.2083	0.7208	0.449	0.7423	0.1817	0.4907	
SIV x estrous cycle phase	0.1506	0.2031	0.8992	0.0967	0.4069	0.0063	0.6047	0.1537
Time of treatment x estrous cycle phase	0.037	0.0121	0.0144	0.0646	0.0005	0.1068	0.891	
ELA x SIV x treatment time	0.2803	0.1868	0.8579	0.1713	0.5834	0.4412	0.9695	
ELA x SIV x estrous cycle phase	0.0056	0.7039	0.7636	0.7116	0.4531	0.225	0.3224	0.5971
ELA x treatment time x estrous cycle phase	0.2111	0.8787	0.3928	0.1144	0.072	0.1472	0.7899	
SIV x treatment time x estrous cycle phase	0.4026	0.02	0.0998	0.1949	0.8512	0.6236	0.9003	

COL1A2 - collagen type 1 α2; COL1 – collagen type I protein; *MMP2* - matrix metallopeptidase 2; *MMP9* - matrix metallopeptidase 9.

Table S2. Listed significant differences of the same treatments between the follicular phase (FP) and mid-luteal phase (MLP) of the estrous cycle, within each treatment time.

Evaluated Variables	Treatment Comparisons	P Value	Figures
COL1A2 transcription	ELA 48h FP *vs* ELA 48 h MLP	*P* < 0.001	1A, 1B
COL1 protein relative abundance	SIV 48h FP *vs* SIV 48 h MLP	*P* < 0.05	1C, 1D
	ELA + SIV 48 h FP *vs* ELA + SIV 48 h MLP	*P* < 0.001	
MMP2 transcription	ELA + SIV 24 h FP *vs* ELA + SIV 24 h MLP	*P* < 0.05	2A, 2B
MMP9 transcription	SIV 24 h FP *vs* SIV 24 h MLP	*P* < 0.01	2C, 2D
	SIV 48 h FP *vs* SIV 48 h MLP	*P* < 0.01	
	ELA 24 h FP *vs* ELA 24 h MLP	*P* < 0.0001	
	ELA + SIV 24 h FP *vs* ELA + SIV 24 h MLP	*P* < 0.0001	
	ELA + SIV 48 h FP *vs* ELA + SIV 48 h MLP	*P* < 0.01	
Pro-MMP-2 activity	ELA 48 h FP *vs* ELA 48 h MLP	*P* < 0.001	3A, 3B
	ELA + SIV 48 h FP *vs* ELA + SIV 48 h MLP	*P* < 0.05	
Active MMP-2 activity	ELA 48 h FP *vs* ELA 48 h MLP	*P* < 0.001	3A, 3B

COL1A2 - collagen type 1 α2; COL 1 – collagen type I; *MMP2* - matrix metallopeptidase 2; *MMP9* - matrix metallopeptidase 9; ELA – elastase; SIV – sivelestat sodium salt; FP – follicular phase; MLP – mid-luteal phase.
